# Naphthalene induced acute kidney injury in an African patient in Ghana: a case report

**DOI:** 10.11604/pamj.2019.32.201.17886

**Published:** 2019-04-24

**Authors:** Elliot Koranteng Tannor, Kojo Awotwi Hutton-Mensah

**Affiliations:** 1 Renal Unit, Department of Medicine, Komfo Anokye Teaching Hospital, Kumasi, Ghana

**Keywords:** Naphthalene, acute kidney injury, acute tubular necrosis, glucose-6-phosphate dehydrogenase deficiency

## Abstract

Naphthalene is commonly used in Ghana as an insecticide and there have been occasional ingestion unintentionally ingestion in children. Naphthalene use has been associated with intravascular haemolysis especially in patients with glucose-6-phoshate dehydrogenase (G6PD) deficiency but its unorthodox use for the treatment of urethritis in a young man and its associated acute kidney injury has not been described in Ghana. This case report bring to fore the indiscriminate use of complementary medicines and the multiple adverse effects on the kidneys after the ingestion of a combination of naphthalene, alcohol and aluminium sulphate (Alum) as a treatment for urethritis upon a friend's suggestion requiring intermittent haemodialysis but recovered completely of his renal functions. Naphthalene ingestion can cause acute tubular necrosis from haemoglobinuria and timely interventions are necessary to restore renal and maintain good renal functions.

## Introduction

Naphthalene is generally used as an insecticide in Ghana. It is mostly toxic when accidentally ingested in children but in adult, it is ingested in suicide attempts [1]. Most countries have banned naphthalene use and replaced it with 1, 4-dichlorobenzene as an insecticide [2] but not in Ghana. Naphthalene is mostly known to cause severe intravascular haemolysis and haemoglobinuria [3] in individuals with glucose-6-phosphate dehydrogenase (G6PD) deficiency and even those with normal levels of G6PD. Intravascular haemolysis has been suggested as a cause of kidney damage by direct toxic injury to the kidneys tubules due to haemoglobinuria [4]. Naphthalene use for the treatment of urethritis has not been described. We therefore set out to describe the case of a young man who was prescribed a combination of naphthalene, alcohol and aluminium sulphate for the treatment of his urethritis by a friend and eventually developed acute kidney injury requiring intermittent haemodialysis from a combination of factors.

## Patient and observation

A 26 year old African male university student with no known medical condition presented to the Emergency Department of the Komfo Anokye Teaching Hospital (KATH) with a 4-day history of jaundice, cola like urine and reduced urine volume associated with palpitation, dizziness and easy fatigability. He had ingested 15 mls twice daily for 2 days a mixture of three naphthalene balls, 100mls of locally brewed ethanol (‘akpeteshie’), olive oil and aluminium sulphate (alum) as self-medication for a urethritis he has suffered a week prior to presentation based on a friends suggestion. He was admitted at a peripheral hospital at the onset of his symptoms for three days and was managed as a case of severe anaemia secondary to intravascular haemolysis from naphthalene containing herbal preparation. He was put on Intravenous (IV) artesunate, IV ceftriazone 2g, Intramuscular (IM) diclofenac 75mg and tablet acetamenophen 1g three times a day then referred to KATH for haemotransfusion and possible haemodialysis when he was noted to have reduced urine output with rising serum creatinine up to 2315umol/L. He arrived at the Emergency Department fully conscious but looked pale, temperature 36.80C, blood pressure 83/60mmHg, pulse rate was 111 beats per minute, respiratory rate was 16 cycle per minute, random blood glucose was 13.6mmol/L, oxygen saturation of 98% in room air, his chest was clinically clear and had no peripheral oedema. His urine was cloudy, cola like with leukocyte (++), protein (++++), blood (++++) on urine dipsticks and urine microscopy revealed pus cells > 30 cells per high power field, red blood cells greater than 12 cells per high power field and motile bacteria (+++) as suggested by his diagnosis of urethritis. His haemoglobin concentration on admission was 5.8g/dl with Mean Corpuscular Volume (MCV) of 86.1fL. Platelet count was 222 x 10^9^/L and white blood cell count was 12.08 x 10^9^/L. His renal function test was deranged with urea of 46.12 mmol/L, creatinine of 2315 umol/L. His serum aspartate transaminase was mildly elevated at 54.9 U/L and had decreased total protein level of 65.1g/L. His screen for sickling status was negative (AA genotype) and was glucose-6-phosphate dehydrogenase deficiency full defect. Serology for Hepatitis B, Hepatitis C and retroviral infection were also negative. An initial diagnosis of: 1) Acute Kidney injury secondary to toxic acute tubular necrosis; 2) Naphthalene induced intravascular haemolytic anaemia; 3) Urethritis/Urinary tract infection were made. He was started on IV 0.9% Normal saline fluids of 3L over 24hours with strict urine output monitoring, IV ciprofloxacin 400mg two times daily, tablet doxycycline 100mg twice daily was prescribed to treat the urethritis and urinary tract infection empirically as cultures were negative. He was haemotransfused with 2 units of packed red blood cells. His urine output for the first 24 hours was 100mls but he was not in pulmonary oedema.

On day 2 of admission, his daily IV fluid input was increased to 3.5L in 24 hours. Urine output increased to 400mls/24hours. Patient however then developed pulmonary oedema which was managed with IV furosemide (80mg twice daily which was increased to 80mg three times daily). Urine microscopy was done by an experienced nephrologist which showed red cell casts, multiple RBCs, muddy brown granular casts. His serum sodium was 125 mmol/L, serum potassium of 5.7mmol/L, serum chloride of 106mmol/L and urine protein creatinine ratio (UPCR) of 1.17g/day. His serum calcium and phosphate were all normal measuring 2.29mmol/L and 1.15mmol/L respectively. Abdominal Ultrasound showed enlarged kidneys bilaterally measuring 12.5cm x 5.6 cm for the left kidney and 14.0 x 6.4cm for the right, echogenic with poor corticomedullary and sinus differentiation. No focal mass, hydronephrosis or calculi were seen. There was also moderate ascites and bilateral pleural effusion which was confirmed to be transudative. His arterial blood gas result revealed a high anion gap metabolic acidosis with pH of 7.194, bicarbonate of 10.8mmol/L and anion gap of 17.1. He was started on 10mls of intravenous (IV) 10% calcium gluconate, salbutamol nebulization 10mg and 5IU of soluble insulin with 100ml of 50% dextrose for hyperkalaemia and sodium bicarbonate 1g three times daily on account of metabolic acidosis. He was also started on IV methylprednisolone 500mg daily for 72 hours then to continue with tab Prednisolone 60mg daily due to suspected acute glomerulonephritis suggested by the presence of red cell cast, dysmorphic red cells and sub-nephrotic proteinuria in preparation for a percutaneous renal biopsy. Despite correction of hyperkalemia, his serum potassium continued to rise to 6.9mmol/L by the 5th day on admission. He was commenced on acute intermittent haemodialysis on account of refractory hyperkalaemia and pulmonary oedema. He had 2 sessions of haemodialysis and urine output improved subsequently to about 1L in 24 hours on steroids. Urine output progressively increased till he was polyuric passing up to 10,950 ml/day which was complicated by hypokalaemia of 2.8mmol/L. This was corrected with oral potassium tablets supplements. Patient's clinical condition improved and his weight over the period reduced from 76.6kg to 56.9kg. Tab prednisolone was slowly tapered down till 5mg daily over 2 months. Repeat urine microscopy showed few RBCs and no casts and there was no indications for biopsy as patient was improving clinically. On discharge after 3 weeks, his urine output was 2700mls and his renal function had normalized with a urea of 6.50mmol/L, creatinine 96umol/L, serum sodium concentration of 139mmol/L, potassium of 4.7mmol/L, Chloride of 103 mmol/L and normocytic anaemia of 9.2g/dl. His weight was 57.0kg. Patient was reviewed after 3 months and found to have a serum creatinine of 94umol/L, haemoglobin of 12.8g/dl with a weight of 58.9Kg.

## Discussion

This is the first ever reported case of naphthalene induced toxic acute tubular necrosis from intravascular haemoglobinuria in Ghana to our knowledge. This case brings to light the dangers of treatment of simple conditions with complementary medicines and the need for thorough investigations for all cases of acute kidney injury (AKI) and the valuable use of urine microscopy in the management of kidney disease in low resource countries like Ghana. In the absence of a percutaneous biopsy, it was not clear what the exact cause of the AKI was but the authors believe it might be a combination of factors with the leading cause being driven mainly by the ingestion of naphthalene and its associated haemoglobinuria in a patient with glucose-6-phosphate deficiency full defect as well as other undeclared contents. Naphthalene is generally used in Ghana as an insecticide but it was strange to find out that some people can prescribe it for the treatment of infections such as urethritis. When naphthalene is ingested intentionally, it is for attempting suicide in adult and mostly accidental ingestion with toxicity in children have been described [1]. Most counties have banned naphthalene use and replaced it with 1, 4-dichlorobenzene as an insecticide [2] but not in Ghana. Naphthalene is mostly known to cause severe intravascular haemolysis and haemoglobinuria [3] in individuals with glucose-6-phosphate dehydrogenase deficiency as found in our patient and even those with normal levels of G6PD. Intravascular haemolysis has been suggested as a cause of kidney damage by direct toxic injury to the kidneys tubules due to haemoglobinuria [4]. They present with cola-like urine, decreasing haemoglobin concentrations in blood and worsening renal functions as was seen in our patient. The confirmation of haemolysis could also be suggested by decreasing serum haptoglobulin elevation and increasing serum lactate dehydrogenase and blood film comments which could not be done due to financial constraints and the fact that diagnosis was clinically made convincingly. The mechanism of haemoglobin induced acute tubular necrosis is believed to be as a result of free haemoglobin filtering at the glomerulus in the dimeric form (one alpha and one beta chain with MW 32 Kda) into the kidneys after serum haptoglobulin is saturated. It binds to the proximal tubular cells via the megalin-cubilin receptors where it leads to direct cytotoxicity of the tubular cells, associated with renal vasoconstriction and also leads to formation of intratubular cast with interaction with Tamm-Horsfall's protein which obstructs the tubules leading to the formation of muddy brown granular cast as seen in our patient [5].

Our patient was also given a non-steroidal anti-inflammatory drug (NSAID) diclofenac at the peripheral hospital which has various effects on the kidney. NSAID have been described as a risk for acute kidney injury through via mechanisms such as ischemic ATN, glomerulopathies, acute interstitial nephritis, and fluid overload [6]. ATN is the most common pathology associated with NSAID and we believe this could synergistically have increased his risk of AKI with the toxic ATN from haemoglobinuria. Our patient also reported with an infection suspected to be a urethritis which he tried treating with the concoction based on a friend's suggestion. Infections are also noted to cause AKI in most patients. The presence of granular cast suggests ATN which could be due to the use of the NSAID diclofenac and or the presence of infection. Acute kidney injury occurs in 19% of patient with moderate infections and up to 51% of patients with severe sepsis with septic shock [7]. Our patient presented with hypotension which increased his risk of AKI. Our patient mixed alcohol with the naphthalene and aluminium sulphate (Alum) and this may have increased the solubility of the substances and increased the absorption as described in literature leading to increased bioavailability and hence increased toxicity [8]. This case report also brings to light the susceptibility of patients to wrong information which may increase their morbidity and mortality. An advice from a friend led the patient to come in with severe AKI and required intermittent haemodialysis to save his life. Globally, 4 billion of the world's population rely on traditional medicine for health care. In Africa, It is known that over 50,000 tons of medicinal plants are consumed annually and over 80% of population in Africa take herbal medication It has been shown that both the rich and poor, educated or uneducated use herbal medications in Ghana as well as most African countries [9]. Aluminium toxicity has been well described in literature as a cause of encephalopathy, osteomalacia and microcytic anaemia especially in patients who have been on chronic dialysis. The use of alum has not been clearly stated as a cause of kidney injury but animal studies have suggested that it could cause injury to proximal tubular cells via oxidative damage [10]. It is still not entirely clear what the cause of the suspected glomerulonephritis was as evidenced by the red cell cast on urine microscopy as there is no clear association with naphthalene, alum or diclofenac in literature. It may be as a result of some undisclosed content in the concoction ingested by our patient. There is the need for further studies to explore glomerulonephritis and its association with the use of complementary medications in Ghana.

## Conclusion

Naphthalene is being used by some people in various combinations for the management of common medical conditions. Naphthalene is a common cause of intravascular haemolysis and haemoglobinuria causing toxic acute tubular necrosis. Our patient's AKI was due to multiple factors that included the haemoglobinuria, infection and the use of NSAIDs. Prompt identification of causes of AKI and management saves lives in low resources countries like Ghana.

## Competing interests

The authors declare no competing interests.
